# Men increase contributions to a public good when under sexual competition

**DOI:** 10.1038/srep29819

**Published:** 2016-07-14

**Authors:** Arnaud Tognetti, Dimitri Dubois, Charlotte Faurie, Marc Willinger

**Affiliations:** 1 Institute for Advanced Study in Toulouse, 21 allée de Brienne, 31015 Toulouse – France; 2 CNRS, UMR 5474, LAMETA, av. R. Dugrand, C.S. 79606, 34960 Montpellier – France; 3 Institute of Evolutionary Sciences, University of Montpellier, CNRS, IRD, EPHE, Montpellier – France; 4 University of Montpellier & Institut Universitaire de France, LAMETA, av. R. Dugrand, C.S. 79606, 34960 Montpellier – France

## Abstract

Why humans cooperate in large groups and with non-kin remains a puzzle for researchers across the natural and social sciences. Investigating whether cooperation is sexually selected could contribute to an understanding of the evolution of human cooperation. Competition for access to mates could indeed select for cooperation. Using controlled laboratory experiments, we analyse whether and how the sex composition of a social environment, testosterone level, and relationship status affect contributions to a public good. The results show that variation in sex composition alters the amount of money that single men (but not men in a couple or women) contribute to a public good. Notably, in line with the competitive helping hypothesis, awareness of the presence of a woman leads to larger contributions by single men, most likely by triggering their competitiveness to be the most cooperative man in the group. However, we find no link between basal testosterone level and cooperativeness. We argue that men, notably single men, adopt cooperative behaviours as a signalling strategy in the context of mate choice and hence that cooperation is partly sexually selected. Our findings highlight the need to consider sexual selection as an additional mechanism for cooperation.

The evolution of cooperation between unrelated individuals remains a challenging issue for evolutionary biologists^1^. The puzzle is that cooperative behaviour is beneficial to recipients but is costly to the actor. Theory suggests that cooperative acts can only be selected if the costs of cooperation are compensated by fitness benefits^2^. Some theoretical^3^,^4^,^5^ and experimental studies^6^,^7^ have shown that sexual benefits could maintain cooperation in a group. Investigations into whether cooperation is sexually selected could thus contribute to our understanding of the evolution of cooperation. Based on controlled and incentivized laboratory experiments, we show that variation in the sex composition of a group of four individuals alters the amount of money that single men (but not men in a couple or women) contribute to a public good, when the sex composition is common knowledge. Notably, in line with the competitive helping hypothesis, awareness of the presence of a woman in the group likely increases single men’s competitiveness in their willingness to appear as the most cooperative individual, leading to larger contributions by those men. We also investigate the potential influence of basal testosterone as a proximate mechanism of this behaviour. However, no association between male testosterone level and contribution to a public good is detected. Despite the absence of testosterone influence on cooperation, we argue that men, especially single men, adopt cooperative behaviours as a signalling strategy in the context of mate choice, and hence that cooperation is – at least partly - sexually selected in humans.

Observations in non-human animal species suggest that sexual selection could be implicated in the evolution of cooperation. Male Lance-tailed Manakins (*Chiroxiphia lanceolata*) perform cooperative efforts, such as singing duets and dancing with other males, which increases their chances of mating with females^8^. In chimpanzees (*Pan troglodytes*), males exchange political support from the dominant male for mating opportunities^9^: dominant males, in turn, tolerate males who support them most frequently in conflicts, with the result that supporters copulate more often than other males.

In humans, there is also evidence that cooperative behaviour toward non-kin could be sexually selected. For example, several studies suggest that cooperative traits are implicated in mate choice^10^,^11^,^12^ and that women are more sensitive to those traits in sexual partners than men are^10^. Cooperative individuals have also been shown to be rated as physically more attractive^13^. Moreover, men seem to use cooperative behaviours as a signalling strategy: in repeated public goods games, men, but not women, have been shown to contribute more to the common account in the presence of opposite sex observers than in the presence of same sex observers or when unobserved^6^,^7^. Moreover, men’s contributions increased across rounds of the public goods game in the presence of female observers, but decreased in the presence of male observers or when unobserved^6^,^7^, a decay pattern that is typically observed in most experiments on voluntary contributions^14^. This increasing pattern of the men’s contributions across rounds was interpreted as competitive cooperativeness (also known as ‘competitive altruism’ or ‘competitive helping’^4^,^5^): men would increase their contributions in response to contributions of other men in order to appear as the most cooperative individual of their group. While theoretical studies demonstrated that competitive cooperativeness could enhance and maintain cooperation^3^,^5^, most empirical studies investigating this possibility offered only suggestive evidence^15^. To our knowledge, only one recent study, using data from online fundraising pages, provided convincing evidence of responsive competitive cooperativeness, i.e. that men compete directly by increasing their cooperativeness in response to displays from competitors^15^. More empirical studies are, therefore, needed to explore the competitive cooperativeness hypothesis. Moreover, most of the studies exploring this hypothesis focused on cooperative interaction in all-male groups where women were only used as observers^6^,^7^. However, cooperative interactions are influenced by the sex of the partners involved^16^, and the sex composition of a social environment has been shown to influence both men’s economic decisions^17^ and a group’s economic performance. For example, the results of a large online business game indicated that groups of two men and one woman displayed higher economic performance compared to other combinations in sex^18^. We, therefore, proposed to examine whether and how the sex composition of a group of four individuals influenced cooperative behaviour, particularly in the context of sexual competition (i.e. in groups involving several men and at least one woman).

If cooperativeness is used by men as a display to attract mates through social reputation and/or prestige, then cooperation might play a major role in mate choice. Cooperative behaviours are costly for the decision-maker^19^. They could therefore signal honestly an individual’s underlying quality (theory of costly signalling)^20^,^21^, such as access to resources or cooperative intent. Cooperative intent could include child care and provisioning^22^,^23^. Indeed, parental investment can be thought as a form of cooperative behaviour, implying a cost for the actor of the cooperative act as well as indirect benefits in the form of improved offspring fitness. Because paternal investment is facultative in humans^24^, women might be sensitive to the cooperativeness of potential mates. Testosterone, the “male” hormone, is a biological marker of both high genetic quality and low paternal investment in human males^25^. We therefore measured the basal level of testosterone (T) of male participants, to examine whether and how T was associated with cooperative behaviour.

Using a self-report personality scale, Harris *et al.*^26^ found that T was positively correlated with aggressiveness and negatively correlated with cooperativeness in both men and women. In addition, high-T men are more likely to reject low offers in ultimatum games^27^. Consistently, men with artificially raised T have been shown to be less generous in ultimatum games, and more likely to punish those who were ungenerous toward them^28^. Hence, men with high-T levels seem more aggressive and less cooperative than low-T men. However, the association between T levels and men cooperativeness could differ according to the type of economics game. Indeed, T is involved in intra-sexual competition and behaviour linked to dominance or status seeking^29^,^30^. In public goods games, T could thus influence the effect of male-male competition on contributions to the public good when females are present^6^,^7^. Hence, there are potentially two opposing predictions about how T levels and contributions to a public good might co-vary. On the one hand, because it has been previously demonstrated that high-T men are less cooperative than low-T men^26^,^27^,^28^, we could expect that high-T men would contribute less. On the other hand, because high-T men are more likely to engage in male-male competition^30^, they could compete with other men by increasing their contributions to the public good, especially in the presence of potential sexual partners. Finally, because T-levels vary as a function of male relationship status (single or in couple)^31^,^32^ and because single men increase more of their attention to potential mates and display more to attract women than men in a couple^33^,^34^, it is also likely that the influence of the presence of women on men’s contributions would be stronger on single men than on men in a couple. We therefore examined how T and relationship status influence men’s contribution to a public good when women are present.

Our experiment was, therefore, designed (i) to test the hypothesis that men attempt to signal their cooperativeness in a context of competitive cooperativeness (i.e. for groups of four individuals, when there are several men and at least one woman in the group) when the group composition is common knowledge and to assess the effect of both (ii) T and (iii) relationship status on contributions to a public good.

Student subjects (n = 320; mean age: 24.1 years ± 0.3 s.e.m.) were recruited to participate in a session of 20 rounds of a standard linear public goods game in groups of four players (see methods section for details). Briefly, at each round, subjects independently decided how to allocate their initial endowment of 20 tokens between a private account and a common account. Allocation decisions yielded payoffs that were measured in points during the experiment and converted into euros at the end of the sessions. Each token allocated by a subject to his/her private account paid off 1 point, while each token contributed to the common account paid off 0.5 point to each of the four group members. At the end of each round, participants were informed about the payoff from their individual account, from the collective account, and they were also informed about the total number of tokens contributed by their whole group to the common account in the current round. Five types of groups that varied in sex composition were constituted in each of our 16 sessions: a group of 4 men, a group of 3 men and 1 woman, a group of 2 men and 2 women, a group of 1 man and 3 women, and a group of 4 women. The groups remained unchanged throughout the duration of session. Before the session, both men and women provided a saliva sample for testosterone assays (although only men samples were analysed) and socio-demographic data such as relationship status (single versus in a couple, either married or not), age and socio-economic status.

Because a wide range of evidence showed that several behavioural and personality traits, including cooperativeness and competitiveness, differ between men and women^16^,^35^, two potentially simultaneous effects had to be distinguished: (i) the intrinsic effect of the players’ sex (contributions to the public good could vary between our five types of groups because men’s and women’s cooperativeness differ and their proportions in these groups are different) and (ii) the effect of knowing the sex of other group members (our main interest here). To disentangle these two effects, two treatments were compared: a control treatment in which participants were not informed about the sex composition of their group (*sex composition concealed*), and a test treatment in which participants were only informed about the sex composition of their group (*sex composition displayed*). In order to perform the *sex composition concealed* treatment, the entire experiment was anonymous: it was run on a computer network and each participant was seated in an individual cubicle containing a computer terminal. Moreover, because facial traits influence individuals’ decisions regarding with whom they cooperate^36^,^37^, participants involved in the eight sessions of the test treatment were informed about the sex composition of their group only by symbols displayed on their screen.

In the test treatment, where the sex composition was displayed, we predicted that contributions to the common account (i) would be larger and (ii) would not decrease across rounds in the groups involving at least two men and at least one woman (a situation favouring *male competitive cooperativeness*) compared to other group compositions (i.e. groups of 4 men and groups of 1 man and 3 women), due to (iii) men’s decisions (but not women’s) and (iv) especially single men’s decisions. Because we investigated *male competitive cooperativeness*, groups of 4 women were not considered in the analyses. However, all-female groups were necessary to prevent uncontrolled effects of gender inequality of the experimental room; hence, a gender-balanced sample of 10 men and 10 women was involved in each session.

We first examined whether the game condition (i.e. displaying the sex composition of the group) influenced a **group’s contribution** to the common account. To that aim, we used linear mixed models (LMM) with the group’s contribution to the common account in a given round as the response variable. Our key explanatory variable was ‘condition’, which involved three categories: (1) the control treatment: ‘*sex composition concealed’* condition; (2) the ‘*male competitive cooperativeness’* condition: sex composition displayed (test treatment) in groups involving at least two men and at least one woman; (3) the ‘*no male competitive cooperativeness*’ condition: sex composition displayed (test treatment) in groups involving only one man or no woman. We also included the variable ‘round’ and its interaction with ‘condition’ to investigate whether the dynamic of the group across rounds was influenced by the game condition. Finally, ‘group ID’ was included as a random factor to account for the repeated measures across rounds.

Secondly, we investigated whether and how the game condition influenced **individual contributions** to the common account for both men and women. Analyses were performed separately for each sex. We used an individual’s contribution to the common account in a given round as the response variable. The key explanatory variable was ‘condition’, as in the previous model. We also included in the model the variable ‘round’ and its interaction with ‘condition’. Moreover, in order to investigate the effects of individual characteristics on contributions, we included in the model the basal T level (for men participants only), relationship status, age, and socio-economic status. Furthermore, to examine whether the effects of individual characteristics on contributions were influenced by the game condition, we included all the interactions between the variable ’condition’ and the other variables. When the interaction between relationship status and game condition was influencing the response variable, we performed the analyses separately for individuals in couple and for single individuals. Finally, ‘individual ID’ nested within ‘group ID’ were included as random factors to take into account for the individual’s repeated measures and their non-independence within groups.

The data were analyzed by multimodel inference^38^,^39^. From a global linear mixed model, a set of all the possible combinations of models was generated, and these models ranked according to their goodness-of-fit to the data based on the Akaike information criterion (AIC)^39^. The difference in AIC (Δ*i*) between the best model (with the lowest *AIC*) and the other models provides a measure of how much more likely the best model is than model *i*. Following Burnham and Anderson^40^, we only considered models with Δ*i* values up to 4. For each model, we calculated a weight (*wi*) as an estimation of the probability that a given model is the best approximating model among this subset of models. We estimate the relative importance of each variable (*I*) by summing the weights *wi* of all models containing this variable. The relative importance of a variable reflects the probability that it is a component of the best model and how it improves the model fit^39^. We computed model-averaged parameters and error estimates for each variable^39^. We also calculated a 95% confidence interval (95% CI) for each variable. When the 95% CI excludes 0, we consider a variable associated with the response variable. Conversely, when the 95% CI includes 0, it indicates that the variable is not systematically linked with higher or lower contribution to the common account and therefore that its effect is not found to be strong. All analyses were conducted using R 3.1.2^41^ and the packages *lme4 (version 1.1-9)*^42^ and *MuMIn (version 1.15.1)*^43^.

## Results

### At the group level

**Figure 1:**
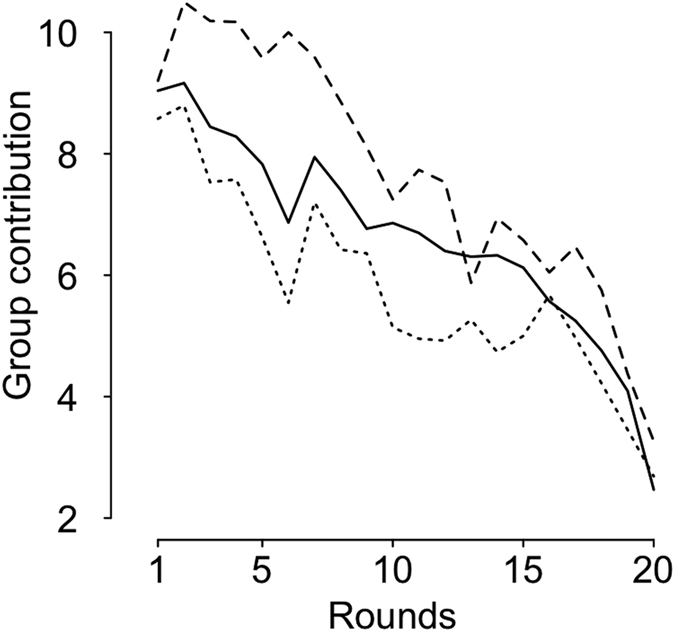
Average contribution to the common account in the three conditions (raw data): *sex composition concealed* (solid line), *no male competitive cooperativeness* (dotted line) and *male competitive cooperativeness* (dashed line).

In line with our predictions, the average contribution to the common account across the 20 rounds seemed to be influenced by the game condition as it was higher in *male competitive cooperativeness* groups (mean ± s.d.: 7.7 ± 2.5) than in *no male competitive cooperativeness* groups (5.8 ± 2.7) or in *sex composition concealed* groups (6.6 ± 2.8). Results of the model selection slightly support the prediction that the game condition influenced a group contribution (*I* = 0.99) as the *male competitive cooperativeness* groups only tended to contribute larger amounts to the common account than did the *no male competitive cooperativeness* groups (β = −8.12, 95% CI [−16.27; 0.03]) when sex composition was displayed (test treatment) (Fig. 1 and Table S1). Moreover, no difference in contributions was observed between *male competitive cooperativeness* groups and *sex composition concealed* groups, given that the 95% CI of the effect included 0 (β = −4.64, 95% CI [−11.53; 2.24]) (Fig. 1 and Table S1). The results of the model selection also revealed that the variable round was likely to strongly influence the group contribution (*I* = 1) as contributions decreased across rounds for each type of group (*no male competitive cooperativeness* groups: β = −1.09, 95% CI [−1.24; −0.94]; *sex composition concealed* groups: β = −1.10, 95% CI [−1.22; −0.99]), even for the *male competitive cooperativeness* groups (β = −1.14, 95% CI [−1.33; −0.95]).

Finally, to confirm that contributions differed between *competitive cooperativeness* groups and the *no male competitive cooperativeness* groups because of the knowledge of the sex of other group members (and not simply because sex affected behaviour and men/women ratios in the groups were different), we compared the contribution of these two types of group when the sex composition was concealed. We therefore performed the same analyses within the *sex composition concealed* groups (i.e. control treatment). As expected, no difference in contributions was found between the *male competitive cooperativeness* groups and the *no male competitive cooperativeness* groups when the sex composition was concealed (β = 2.09, 95% CI [−5.44; 9.61], *I* = 0.83).

### At the individual level

As the interaction between men’s relationship status and game condition was likely to strongly influence a group contribution (*I* = 1), we therefore analysed separately men in a couple and single men. Overall, and in line with our predictions, the analyses revealed that the game condition seemed to influence single men’s contributions, but very weakly those of men in a couple (Tables S2 and S3).

**Figure 2:**
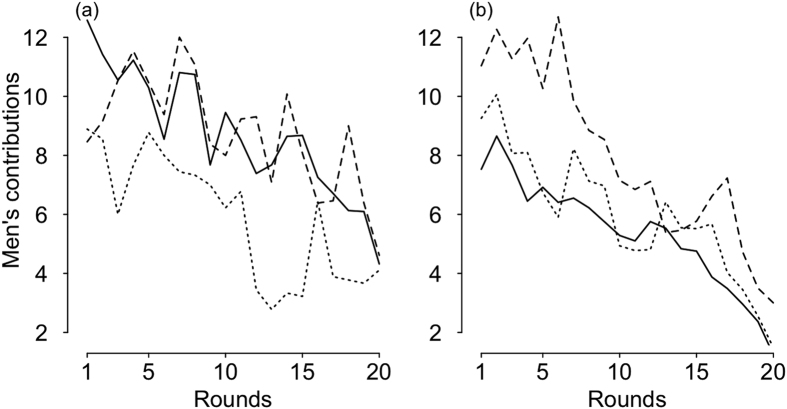
Men’s in a couple (**a**) *versus* single men’s (**b**) average contribution to the common account in the three conditions (raw data): *sex composition concealed* (solid line), *no male competitive cooperativeness* (dotted line) and *male competitive cooperativeness* (dashed line).

Indeed, contributions of men in a couple were unlikely to depend on the game condition (*I* = 1) (Fig. 2a and Table S2): contributions in *male competitive cooperativeness* groups were neither different from those in *no male competitive cooperativeness* groups (β = −0.69, 95% CI [−5.93; 4.56]) nor from those in *sex composition concealed* groups (β = 3.09, 95% CI [−1.04; 7.23]), given that CI fully overlapped 0 (Table S2). Moreover, contributions decreased across rounds (*I* = 1) among all groups (*male competitive cooperativeness* groups: β = −0.24, 95% CI [−0.35; −0.13]; *no male competitive cooperativeness groups*: β = −0.29, 95% CI [−0.36; −0.22]; *sex composition concealed groups*: β = −0.47, 95% CI [−0.53; −0.40]).

On the contrary, the game condition was likely to strongly influence single men’s contributions (*I* = 1): single men in *male competitive cooperativeness* groups contributed larger amounts to the common account than those in *no male competitive cooperativeness* groups (β = −3.98, 95% CI [−6.40; −1.56]) or *sex composition concealed* groups (β = −5.08, 95% CI [−7.39; −2.77]), given than CI did not include 0 (Fig. 2b and Table S3). However, contributions decreased across rounds (*I* = 1) among all groups (*no male competitive cooperativeness groups*: β = −0.33, 95% CI [−0.40; −0.26]; *sex composition concealed groups*: β = −0.28, 95% CI [−0.35; −0.22]) even for *male competitive cooperativeness* groups (β = −0.47, 95% CI [−0.55; −0.39]).

Contrary to our predictions, we did not find any evidence that testosterone level influenced contributions: the 95% CI of the effect of T included 0 for both single men (β<0.0001, 95% CI [−0.002; 0.003], *I* = 0.01) and men in a couple (β<0.0001, 95% CI [−0.01; 0.01], *I* = 0.01) (Tables S2 and S3). Similarly, the 95% CI of the effect of the interaction between the T level and the game condition included 0 for both types of men (see Tables S2 and S3 for details), suggesting that the potential influence of T did not vary with the game condition. Finally, no difference of basal T-level (Student’s t-test: t(85) = 0.99, *p* = 0.33) between single men (mean ± s.d.: 103.41 ± 38.16 pg/ml) and men in couple (96.55 ± 37.77 pg/ml) was detected.

**Figure 3:**
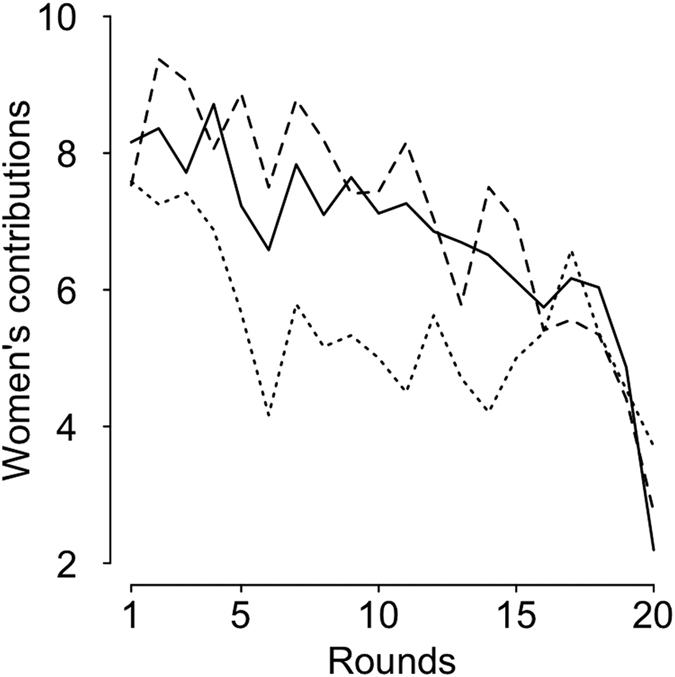
Women’s average contribution to the common account in the three conditions (raw data): *sex composition concealed* (solid line), *no male competitive cooperativeness* (dotted line) and *male competitive cooperativeness* (dashed line).

Women’s contributions were unlikely to depend on the game condition (*I* = 0.73): their contributions in *male competitive cooperativeness* groups were neither different from those in *no male competitive cooperativeness* groups (β = −1.12, 95% CI [−3.84; 1.61]), nor than those in *sex composition concealed* groups (β = −0.39, 95% CI [−2.31; 1.53]) given that the CI fully overlapped 0 (Fig. 3 and Table S4). Women’s contributions decreased across rounds (*I* = 1) among all groups (*male competitive cooperativeness* groups: β = −0.19, 95% CI [−0.23; −0.15]; *no male competitive cooperativeness groups*: β = −0.19, 95% CI [−0.26; −0.12]; *sex composition concealed groups*: β = −0.19, 95% CI [−0.26; −0.12]). No effect of relationship status on women’s contributions was detected (β = 0.05, 95% CI [−0.49; 0.59], *I* = 0.1). Moreover, the interaction between the game condition and relationship status was absent from all considered models with a Δi<4, indicating that relationship status was unlikely to influence women’s contributions across conditions.

Finally, we did not find any evidence that an individual’s age and its interaction with game condition influenced contributions for men or women as the 95% CI of their effect included 0 (Tables S2 to S4). Similarly, no effect of the socio-economic status was found (Tables S2 to S4), and its interaction with game condition was absent from the subset of best models for both single men and women (we did not test the interaction between the socio-economic status and game condition for men in couple due to unbalanced data).

## Discussion

Despite a large amount of theoretical and experimental investigations, human cooperation in large groups and between non-kin remains a puzzle for evolutionary researchers. Investigating whether cooperation is sexually selected could contribute to understanding the evolution of cooperation. Here, we used a controlled laboratory experiment to test whether the awareness of the presence of potential mates induces men to compete for who is the most cooperative man in a group. Using groups of four individuals varying in their sex composition, our experimental design tested whether groups containing several men and at least one woman would exhibit higher level of cooperativeness than other types of group in a standard linear public goods game. We also investigated whether and how testosterone levels and relationship status affected men’s contributions to a public good when they were informed about the presence of potential mates.

We found some evidence supporting the competitive cooperativeness hypothesis. Contributions to the public good of groups involving several men and at least one woman tended to be larger than those of groups involving only one man or no women. More importantly, we found that single men (but not men in a couple or women) contributed more to the public good when they were aware of the presence of at least one another man and another woman in their group. These findings demonstrate that the social environment influences single men’s cooperativeness and that awareness of a woman’s presence is likely to induce men to compete to be the most cooperative individual in the group.

We hypothesised that men, notably those who are single, would cooperate more to signal their cooperativeness to women because it improves their reputation. Indeed, men have been previously found to cooperate when their reputation is involved^6^,^7^,^44^. Moreover, studies have shown that generosity increases social status^45^ and that reputation as a cooperative individual positively impacts attractiveness^46^. Several experimental studies have also highlighted the importance of cooperative traits in mate choice and that men are more cooperative and generous in the presence of women^7^,^10^,^11^,^13^,^44^,^47^.

However, under the competitive helping hypothesis we would expect an increase of contributions by men across rounds. Previous studies^6^,^7^ showed that men’s contributions increased or were stable across 5 successive rounds of a public goods game when men’s identity and contributions were observable by a woman sitting next to them. However, they decreased if men were observed by a man or when unobserved, which is a decay pattern that is typically observed in most experiments on voluntary contributions^14^. Our experimental data also exhibited such decay in all group types. This may seem to contradict the competitive cooperativeness hypothesis according to which men make larger contributions than other members of the group when they know that a woman is present, leading to a stable or increasing pattern of contributions. However, two important observations support the competitive cooperativeness hypothesis in our experiment.

First, single men’s contributions in *male competitive cooperativeness* groups seem stable during the first six rounds but decrease afterwards (Fig. 2b), pointing out the importance of the non-anonymity in the maintenance of group cooperation. Indeed, compared to previous studies, our study did not enable men to show off directly in front of women: subjects played 20 rounds of the constituent game and were assigned to isolated cubicles without the possibility to communicate or observe individuals (including women) with whom they interacted. They were informed about the presence of women in their group only by symbols, and all contributions were anonymous. Even though, single men seemed to display and signal their cooperativeness by allocating larger contributions suggesting that a kind of audience-like effect was activated. We therefore hypothesized that awareness of a woman’s presence by a symbol triggers single men’s competitiveness in early rounds of the repeated game. Indeed, its effect led to single men’s larger contributions even if it was not strong enough to maintain male competition and prevent the decay of contributions. Follow-up studies could examine whether single men’s contributions in the *male competitive cooperativeness* groups would be sustainable when the anonymity of individuals’ contributions and their identity are relaxed.

Second, and more importantly, observations suggest that men in *competitive cooperativeness* groups compete with each other by “beating the previous score”, i.e. by contributing more to the common account in the current round than the average contribution of the three other group members in the previous round (Fig. S1a). However, in this type of group, women’s contributions were lower than the average contribution of other members in the previous round (Fig. S1a). Thus, it is likely that women caused a decay pattern that, in turn, decreased men’s contributions in later rounds. To test this hypothesis we performed linear mixed models. The response variable was the difference between an individual’s contribution to the common account in a current round and the average contribution of the three other group members in the previous round. The results showed that in the *male competitive cooperativeness* groups (Table S5 and Fig. S1a), men did contribute more to the common account than the average contribution of others in the previous round (β = 1.42, 95% CI [0.18; 2.65]). However, this difference between their contributions and the average contribution of others in the previous round decreased across rounds (β = −0.12, 95% CI [−0.19; −0.04]). Women on the contrary behaved as imperfect conditional contributors: they contributed less than the average contribution of the other members in the previous round (β = −1.69, 95% CI [−3.28; −0.09]) across all rounds (β = 0.06, 95% CI [−0.04; 0.16]) (Table S5 and Fig. S1a). In the *no male competitive cooperativeness* groups, no difference was found between an individual’s contribution in the current round and the average contribution of the other members in the previous round for both men and women (Table S5 and Fig. S1b). Overall, these results support the conjecture that awareness of a woman’s presence in the group induced men to compete in early rounds: as predicted by the competitive cooperativeness hypothesis, men in the *male competitive cooperativeness* groups seem to increase their contributions to the common account in response to the contributions of the other members of the group in the previous round. However, the decay dynamic introduced by women behaving as imperfect conditional contributors likely influenced men to decrease their competitive efforts in later rounds, leading them towards a conditionally cooperative behaviour.

We also tested the *female competitive cooperativeness hypothesis*, by comparing groups with several women and at least one man (i.e. groups of 2 women and 2 men and groups of 3 women and 1 man) versus all-female groups (i.e. 4 women) and groups with only one woman. However, we found no difference between groups and treatments (results available upon request).

This behavioural difference between men and women or between men in a couple and single men triggered by the sex composition of their group could be due to hormonal mechanisms or a cognitive bias. However, we found no difference of basal T-level between single men and men in couple. More importantly, no effect of basal T on men’s contributions was found neither, even though T is known to mediate male mating strategies^25^,^48^ and influence cooperativeness and competitiveness^26^,^27^,^28^. Previous studies showed that men’s competitiveness is related to variations in T level and not to basal T^49^, and a woman’s presence is associated with an increase of T levels, competitiveness and risk taking in men^50^,^51^,^52^. Therefore, follow-up studies could examine whether single men’s larger contributions in the *male competitive cooperativeness* groups are linked to an increase of the T-level due to the presence of a woman and their willingness to appear as the most altruistic man in the group.

Another possible explanation for the absence of a relationship between baseline T and cooperativeness could be due to a confounding effect of cortisol levels. Indeed, it has been recently shown that cortisol levels influence the relationship between T and behaviour (dual hormone hypothesis): a high T/low cortisol profile was found to be linked with high levels of competitiveness^53^, dominance^53^, and lower empathy scores^54^ while a high T/high cortisol profile was linked with low competitiveness and high empathy scores^53^,^54^. In other words, a high-T profile might be predictive of both high and low competitive dispositions depending on the concomitant cortisol levels.

Moreover, we cannot rule out the possibility that symbols displaying the sex composition (compared to the presence of women) are insufficient to trigger a different behavioural response between high and low T men. However, as the sex composition of the group differently influences men (notably single ones) and women, it is likely that a cognitive bias or neurochemical system may be involved. For example, a recent study demonstrated that the polymorphism in the monoamine oxidase A gene (MAOA) (coding for an enzyme responsible for the degradation of neurotransmitters such as serotonin and dopamine) influenced men’s contributions in a public goods game depending on their social environment^55^. Similar effects have also been found between cooperation and the polymorphism of the dopamine D4 receptor (DRD4) gene^56^. Therefore, similar mechanisms could be involved in our study to explain the effect of the social environment on men’s contributions to a public good.

Our results also shed new light on the relationship between cooperativeness and one’s sex. We provide new evidence that differences in cooperativeness between the sexes are context-dependent. Sex differences in cooperativeness has to date been examined in several economic experiments. However, the available evidence is mixed: some experiments found a higher cooperativeness of men^57^,^58^, of women^59^,^60^ or no differences between sexes^61^,^62^. These experiments differed in many respects, including in their methods, subject pools and social dilemma games. A meta-analysis by Balliet *et al.*, based on 272 experiments on social dilemmas generated over 50 years, showed that there is no difference in cooperativeness between women and men in general^16^. Moreover, they pointed out that several factors influence the association between sex and cooperation, such as the sex of the partners involved in the interactions, or the duration of the interactions. For example, there is more cooperation in male-male interactions than in female-female interactions, and men are more cooperative than women in repeated interactions. In addition, women are more cooperative than men when the voluntary contribution game is negatively framed (i.e. when each token invested in the private good represents an opportunity cost to the other group members) rather than positively framed^63^. Finally, women cooperate more than men in the presence of members of their social group, but men cooperate more than women in the presence of strangers^64^. Similarly, our data show that the sex composition of groups affects the relative contribution of men (notably single men) compared to women. When the sex composition of the group is displayed, single men contribute more to a public good in groups constituted of several men and at least one woman. Our finding therefore supports the conjecture that cooperativeness of men and women is highly context-dependent and demonstrates the need for controlling for sex composition in economic experiments designed for studying cooperative behaviour, a factor that is often overlooked.

Finally, our study provides new insights on the audience effect on cooperative behaviour. It is already well-known that when individual contributions are identifiable, the so-called “audience effect” triggers higher contributing efforts, both in women and in men^65^,^66^ but does not prevent the decay of the group contribution when the voluntary contribution game is played repeatedly^65^. However, when men’s contributions are observed by a female audience, group cooperation is sustainable^6^ and in some cases even leads to increasing group contributions^7^. In our study, single men’s cooperativeness was affected by the sex composition of their group even in the absence of any observers, which suggests that a kind of audience-like effect is activated. The mere knowledge of the sex composition of the group through symbols caused striking modifications of cooperative behaviour. Behaviours related to audience effect, which have been selected in natural ecological settings, are - somehow inappropriately - expressed here despite the anonymity of this experimental setting. It is unlikely that humans have been selected to deal with anonymous situations or other laboratory conditions, which have been unusual or absent throughout human evolutionary history. Nevertheless, these experiments are useful because of what they reveal about the behavioural, psychological and cognitive processes and biases that have been selected for in natural ecological settings^1^,^67^,^68^. Hence, behaviour in the laboratory has to be explained by considering the environment in which that behaviour evolved^1^. We therefore suggest that the cognitive responses to the sex composition found here, in laboratory conditions, evolved because of their positive consequences on access to mates in the real world setting, and a mechanism able to silence these responses in an anonymous context did not have any chance to evolve.

To sum up, the key result of our study is that variation in the sex composition of the social environment affects single men’s, but not men’s in a couple or women’s, contributions to a public good. The awareness of the presence of a woman leads to larger contributions by single men, most likely by triggering their competitiveness to be the most cooperative men in the group. We therefore argue that men, notably those who are single, use cooperative behaviours as a signalling strategy in the context of mate choice and thus that cooperation can be partly sexually selected. However, investigations on the role of sexual selection in the evolution of cooperation is rare in non-human social species^69^,^70^,^71^ which limits the interpretation of our results in a broader cross-species context. Our study provides new insights into the understanding of the evolution of cooperation in humans and stresses the need to investigate the potential role of sexual selection in the evolution of cooperation in other social species. Our findings also have practical implications for human resources management and for personal economics, as well as for the design of non-monetary group incentives.

## Methods

The methods were carried out in accordance with the approved guidelines. The protocols used to recruit participants and to collect data were approved by the French national committee of informatics and liberties (CNIL declaration # 1321739). Written informed consent was obtained from all participants.

### Protocol

The experiment was conducted in 2012 at the LEEM, the experimental laboratory of the LAMETA (Department of Economics, University of Montpellier, France). The participants were recruited from a pool of 7000 volunteers from various fields of study at the University of Montpellier in France. None of them had ever participated in a public goods game experiment.

The experiment was run on a computer network (the program has been developed using LE2M - Software for the Economic Experiments of Montpellier developed by D. Dubois and JM. Rousselle) and was broken down into 16 sessions all beginning at 2 p.m. In order to prevent uncontrolled effects of gender inequality, each session involved a gender-balanced sample of 20 participants (10 men and 10 women). To prevent visual contact, each participant was seated in an individual cubicle containing a computer terminal. Communication was not allowed. At the beginning of each session, participants had to read a written copy of the instructions. Then the instructions were read aloud by a research assistant in order to implement common knowledge of the game and the tasks (instructions available upon request at dimitri.dubois@lameta.univ-montp1.fr). Questions were allowed and answered privately. Then, participants’ understanding of the instructions was checked by a computerised questionnaire. To guarantee experimenter-subject and subject-subject anonymity, a subject number was assigned to each participant.

### Experimental design

In each session, participants played 20 rounds of a standard linear public goods game. At the beginning of each round, subjects independently decided how to allocate their initial endowment of 20 tokens between a private account and a common account. Allocation decisions yielded payoffs that were measured in points during the experiment and converted into euros at the end of the sessions. Each token allocated by a subject to his/her private account paid off 1 point while the common account paid off 0.5 point (marginal per capita return = 0.5). It was made clear that each token allocated to the common account would provide exactly the same payoff to each of the four group members regardless of who contributed that token.

At the end of each round, participants were informed about the payoff from their individual account and from the collective account. They were also informed about the total number of tokens contributed by their group to the common account in the current round. At any time, subjects could also access a history screen that displayed a table reporting the following information for the past periods: the period number, the player’s contribution to the private account and to the common account, the total amount contributed by his/her group to the common account, his/her payoff for the corresponding period, and his/her cumulative payoff since the beginning of the game.

Subjects were aware that the game would end after the 20^th^ round and that their accumulated payoff would be converted into euros according to the conversion rate 1 euro for 30 points. Each subject was paid in private.

### Demographic data

After the final round, socio-demographic data was collected for each participant: age, socio-economic status and relationship status (single versus in a couple, either married or not).

### Saliva collection and T assays

T levels were measured in saliva samples. This non-invasive technique has been previously validated and yields T levels that are highly correlated with serum levels. At the start of the experiment, each male or female subject was asked to rinse his/her mouth with fresh water and to wait 5 minutes. Then he/she was given one labelled tube and straw (Salicaps kits, IBL-Hamburg) to collect saliva. Because the change of T rate in the diurnal cycle is the lowest in the early afternoon^72^, saliva samples were collected at 2.00 p.m. Samples were kept cold during the duration of the experiment then stored at −80 °C. T levels, from male samples only, were analyzed by Luminescence ImmunoAssay (LIA) technique, using LIA Testosterone kits (IBL, Hamburg). The assay of each sample was replicated twice and only measures whose inter-assay CV was lower than 10% were used.

## Additional Information

**How to cite this article**: Tognetti, A. *et al.* Men increase contributions to a public good when under sexual competition. *Sci. Rep.* **6**, 29819; doi: 10.1038/srep29819 (2016).

## Supplementary Material

Supplementary Information
