# Supplementary material for: Men increase contributions to a public good when under sexual competition
Source: Sci Rep. 2016 Jul 14;6:29819. doi: 10.1038/srep29819 (PMC4944141; doi:10.1038/srep29819)

Men increase contributions to a public good when under sexual competition

Arnaud Tognetti, Dimitri Dubois, Charlotte Faurie & Marc Willinger

Supplementary data: Tables S1 to S5 and Figure S1.

Table S1. The effect of game condition on group contribution to the common account ($n_{observation}$ = 1280 from 64 groups). Linear mixed models were used and analysed by multimodel inference (see manuscript for more details). The relative importance of each variable is given. For categorical variables, estimate and 95% confidence interval (95% CI) represent the relative estimate compared to the reference category (underlined term).

Amount contributed to the common account

	Relative importance	Estimate [95% CI]
Intercept		42.75 [37.01; 48.48]
Condition	0.99	
No male competitive coop. vs. Male competitive coop.		-8.12 [-16.27; 0.03]
Sex composition concealed. vs. Male competitive coop.		-4.64 [-11.53; 2.24]
Round	1	-1.14 [-1.33; -0.95]
Interaction Condition x Round	0.16	
No male competitive coop. vs. Male competitive coop. x Round		0.05 [-0.21; 0.31]
Sex composition concealed vs. Male competitive coop. x Round		0.04 [-0.16; 0.24]

Table S2. The effect of game condition and individual characteristics on contributions to the common account of men in a couple ($n_{observation}$ = 880 from 44 men divided in 29 groups). Linear mixed models were used and analysed by multimodel inference (see manuscript for more details). The relative importance of each variable is given. For categorical variables, estimate and 95% confidence interval (95% CI) represent the relative estimate compared to the reference category (underlined term).

Amount contributed by men in couple to the common account

	Relative importance	Estimate [95% CI]
Intercept		10.80 [7.27; 14.32]
Condition	1	
No male competitive coop. vs. Male competitive coop.		-0.69 [-5.93; 4.56]
Sex composition concealed. vs. Male competitive coop.		3.09 [-1.04; 7.23]
Round	1	-0.24 [-0.35; -0.13]
Age*	0.95	-0.90 [-4.59; 2.78]
SES	0.26	
Middle vs. Low		0.18 [-1.46; 1.81]
High vs. Low		0.31 [-1.58; 2.20]
Testosterone	0.01	<0.0001 [-0.01; 0.01]
Interaction Condition x Round	1	
No male competitive coop. vs. Male competitive coop. x Round		-0.05 [-0.21; 0.11]
Sex composition concealed vs. Male competitive coop. x Round		-0.23 [-0.36; -0.09]
Interaction Condition x Age	0.56	
No male competitive coop. vs. Male competitive coop. x Age		-0.53 [-5.01; 3.94]
Sex composition concealed vs. Male competitive coop. x Age		-0.33 [-4.11; 3.45]
Interaction Condition x Testosterone	0.01	
No male competitive coop. vs. Male competitive coop. x T		<0.0001 [-0.0002; 0.0002]
Sex composition concealed vs. Male competitive coop. x T		<0.0001 [-0.0007; -0.0007]

* the variable ‘age’ was centered and scaled.

Table S3. The effect of game condition and individual characteristics on single men's contributions to the common account ($n_{observation}$ = 1820 from 91 men divided in 50 groups). Linear mixed models were used and analysed by multimodel inference (see manuscript for more details). The relative importance of each variable is given. For categorical variables, estimate and 95% confidence interval (95% CI) represent the relative estimate compared to the reference category (underlined term).

Amount contributed by single men to the common account

	Relative importance	Estimate [95% CI]
Intercept		13.14 [11.27; 15.01]
Condition	1	
No male competitive coop. vs. Male competitive coop.		-3.98 [-6.40; -1.56]
Sex composition concealed. vs. Male competitive coop.		-5.08 [-7.39; -2.77]
Round	1	-0.47 [-0.55; -0.39]
Age*	1	-1.11 [-2.27; 0.04]
SES	0.56	
Middle vs. Low		-0.30 [-1.66; 1.06]
High vs. Low		0.38 [-1.14; 1.91]
Testosterone	0.01	<0.0001 [-0.002; 0.003]
Interaction Condition x Round	1	
No male competitive coop. vs. Male competitive coop. x Round		0.14 [0.03; 0.25]
Sex composition concealed vs. Male competitive coop. x Round		0.19 [0.08; 0.29]
Interaction Condition x Age	0.84	
No male competitive coop. vs. Male competitive coop. x Age		-0.22 [-1.65; 1.21]
Sex composition concealed vs. Male competitive coop. x Age		1.21 [-0.65; 3.07]
Interaction Condition x Testosterone	0.01	
No male competitive coop. vs. Male competitive coop. x T		<0.0001 [-0.0001; 0.0001]
Sex composition concealed vs. Male competitive coop. x T		<0.0001 [-0.00009; 0.00009]

* the variable 'age' was centered and scaled.

Table S4. The effect of game condition and individual characteristics on women's contributions to the common account ($n_{observation}$ = 1860 from 93 women divided in 48 groups). Linear mixed models were used and analysed by multimodel inference (see manuscript for more details). The relative importance of each variable is given. For categorical variables, estimate and 95% confidence interval (95% CI) represent the relative estimate compared to the reference category (underlined term).

Amount contributed by women to the common account

	Relative importance	Estimate [95% CI]
Intercept		8.76 [7.01; 10.51]
Condition	0.73	
No male competitive coop. vs. Male competitive coop.		-1.12 [-3.84; 1.61]
Sex composition concealed. vs. Male competitive coop.		-0.39 [-2.31; 1.53]
Round	1.00	-0.19 [-0.23; -0.15]
Age*	0.62	-0.42 [-1.42; 0.58]
SES	0.10	
Middle vs. Low		0.08 [-0.63; 0.80]
High vs. Low		0.02 [-0.60; 0.64]
Relationship status	0.10	0.05 [-0.49; 0.59]
Interaction Condition x Round	0.05	
No male competitive coop. vs. Male competitive coop. x Round		0.006 [-0.05; 0.06]
Sex composition concealed vs. Male competitive coop. x Round		0.002 [-0.02; 0.03]
Interaction Condition x Age	0.57	
No male competitive coop. vs. Male competitive coop. x Age		0.03 [-1.01; 1.06]
Sex composition concealed vs. Male competitive coop. x Age		0.97 [-0.96; 2.91]

* the variable 'age' was centered and scaled.

Table S5. The effect of an individual's sex on the difference between his/her contribution to the common account in a round t and the average contribution of the three other group members in the previous round (a) for the *male competitive cooperativeness* groups and (b) the *no male competitive cooperativeness* groups ($n_{observation}$ = 1216 - 64 groups for both *male competitive cooperativeness* and *no male competitive cooperativeness* groups). Linear mixed models were used and analysed by multimodel inference (see manuscript for more details). The relative importance, estimate and 95% confidence interval (95% CI) of each variable are given.

a. Difference in contributions in *male competitive cooperativeness* groups

	Estimate [95% CI]
Sex	
Men	1.42 [0.18; 2.65]
Women	-1.69 [-3.28; -0.09]
Round	-0.12 [-0.19; -0.04]
Sex x round	0.18 [0.06; 0.30]

No relative importance is given because only one model was present after the model selection procedure.

b. Difference in contributions in *no male competitive cooperativeness* groups

	Relative importance	Estimate [95% CI]
Sex	0.37	
Men		-0.05 [-0.82; 0.72]
Women		-0.23 [-1.37; 0.92]
Round	0.01	-0.0001 [-0.006; 0.006]

Figure S1. Average contribution to the common account (a) in the *male competitive cooperativeness* condition and (b) in the *no male competitive cooperativeness* condition (raw data): men (solid line), women (dashed line) and average contribution of the other members of the group in the previous round (dotted line).

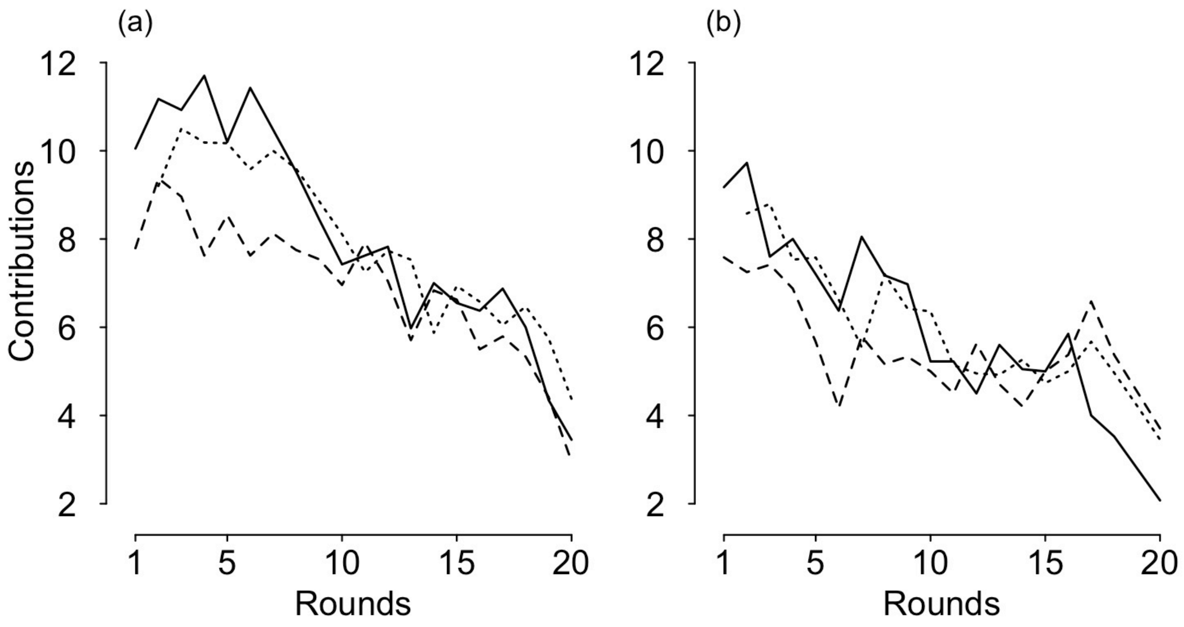

Supplement: Supplementary Information [file srep29819-s1.pdf]
